# Fighting Asian Soybean Rust

**DOI:** 10.3389/fpls.2016.00797

**Published:** 2016-06-07

**Authors:** Caspar Langenbach, Ruth Campe, Sebastian F. Beyer, André N. Mueller, Uwe Conrath

**Affiliations:** ^1^Department of Plant Physiology, RWTH Aachen UniversityAachen, Germany; ^2^BASF Plant Science Company GmbHLimburgerhof, Germany

**Keywords:** Asian soybean rust, *Phakopsora pachyrhizi*, fungicide insensitivity, host resistance, non-host resistance, plant breeding, plant biotechnology

## Abstract

*Phakopsora pachyrhizi* is a biotrophic fungus provoking SBR disease. SBR poses a major threat to global soybean production. Though several *R* genes provided soybean immunity to certain *P. pachyrhizi* races, the pathogen swiftly overcame this resistance. Therefore, fungicides are the only current means to control SBR. However, insensitivity to fungicides is soaring in *P. pachyrhizi* and, therefore, alternative measures are needed for SBR control. In this article, we discuss the different approaches for fighting SBR and their potential, disadvantages, and advantages over other measures. These encompass conventional breeding for SBR resistance, transgenic approaches, exploitation of transcription factors, secondary metabolites, and antimicrobial peptides, RNAi/HIGS, and biocontrol strategies. It seems that an integrating approach exploiting different measures is likely to provide the best possible means for the effective control of SBR.

## Introduction

**FIGURE 1:**
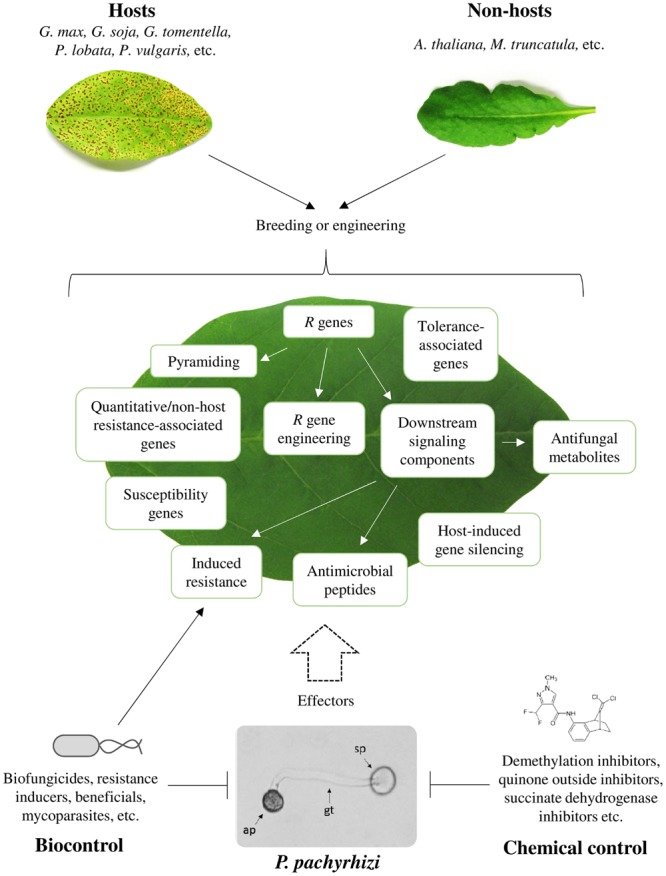
**Strategies for controlling SBR.** Exploitation of different genetic resources (host and non-host plants), biocontrol agents, and chemical fungicides to combat *Phakopsora pachyrhizi*. ap, appressorium; gt, germ tube; sp, uredospore.

SBR is currently the most severe soybean (*Glycine max*) disease. SBR is caused by *Phakopsora pachyrhizi*. The biotrophic basidiomycete threatens soybean production all over the globe, but the threat is most severe in the major soybean growing areas in South America. In Brazil SBR has caused crop losses of more than US$ 10 billion since its first endemic outbreak in 2001 (Yorinori et al., 2005; da Silva et al., 2014). Currently, three major strategies serve to manage SBR (**Figure 1**). First, applying chemical fungicides. Second, breeding or engineering of SBR-resistant soybean cultivars, and third, employing specific cultivation practices, such as planting early ripening varieties, monitoring fields, eliminating secondary hosts, and introducing soybean-free growth periods (60–90 days) in the threatened areas (Hartman et al., 2005; Godoy, 2011; Kendrick et al., 2011). Here, we elaborate on these strategies, and we also discuss the potential of AMPs, RNAi/HIGS, and biocontrol measures for controlling SBR. A detailed description of the life cycle, host range and distribution of *P. pachyrhizi* has been provided earlier (Goellner et al., 2010).

### Chemical Control of SBR

Fungicide use is the most effective means for controlling SBR these days. In Brazil, at least three fungicide applications are needed per season thus raising costs of ∼US$2 billion for soybean disease control annually (Godoy et al., 2015). In contrast to multisite fungicides (e.g., mancozeb) with comparatively low performance, the DMI and the QoI classes of fungicide are prime chemicals for fighting *P. pachyrhizi*. Since 2013, fungicides of the highly active SDHI class are available for SBR control (Guicherit et al., 2014). Because this new fungicide class performs extraordinarily well, the number of available SDHI fungicides and the intensity of their use is likely to steadily increase over the next couple of years (Godoy et al., 2015). However, the excessive use of fungicides increases the chance of fungal strains with evolved insensitivity to the fungicides in use. In the recent past, this was true for the azole-class fungicides to which *P. pachyrhizi* and other fungal pathogens have become insensitive (Godoy, 2012). The FRAC assigned rust fungi, including *P. pachyrhizi*, to the low-risk group of fungi (Brent and Holloman, 2007). However, *P. pachyrhizi* and other causes of polycyclic plant diseases are highly likely to evolve fungicide insensitivity because of the high number of spores they produce (Bradley, 2007).

The mechanism of fungal insensitivity to DMIs is highly complex and variable. After several years of fungicide use, a significant reduction in DMI efficacy to *P. pachyrhizi* was detected in Brazil (Scherm et al., 2009; Barbosa et al., 2013; Reis et al., 2015). The insensitivity is caused either by point mutations in the fungal *cyp51* gene or by *cyp51* overexpression (Schmitz et al., 2014). The major mechanism of QoI and SDHI insensitivity is by point mutations in the *cyt b* and *sdh b/c/d* genes, respectively. These mutations were reported for many plant-pathogenic fungi (Kim et al., 2003; Grasso et al., 2006a; Sierotzki et al., 2007; Sierotzki and Scalliet, 2013). The most common mutation for QoI insensitivity [substitution of glycine to alanine at position 143 of Cyt b] was not yet detected in rusts probably because of presence of a type-I intron after codon 143 (Grasso et al., 2006a,b,c; Oliver, 2014; Klosowski et al., 2015). Nucleotide substitutions in this codon would prevent intron splicing thus leading to a defective Cyt b protein (Grasso et al., 2006a). However, another *cyt b* mutation (F129L) was reported to confer QoI insensitivity in various fungi including *P. pachyrhizi* (Leiminger et al., 2014; Klosowski et al., 2015). For *P. pachyrhizi* SDHI insensitivity was not reported yet. However, the increased use of SDHIs is likely to further enhance the selection pressure for SDHI insensitivity in *P. pachyrhizi* (Godoy et al., 2015). MDR, as reported for *Botrytis cinerea* and other fungi (Kretschmer et al., 2009) also was not observed in *P. pachyrhizi* so far. To assess the risk and impact of fungicide-insensitive isolates, we recommend generating insensitive fungal mutants in the laboratory. Investigating such mutants is likely to disclose mechanisms underlying fungicide insensitivity, enable recommendations for avoiding selection of insensitive fungal populations, and developing novel mode-of-action fungicides. Applying fungicides preventively or as early as possible in the diseases cycle before or shortly after *P. pachyrhizi* infection is crucial for effective SBR control (Mueller et al., 2009; Godoy, 2012). Therefore, early SBR detection and precise forecasts are required for efficient SBR disease management.

Probably the best and most sustainable control of SBR is by providing soybean genotypes resisting *P. pachyrhizi* (see below). Growth of SBR resistant genotypes is likely to be associated with reduced fungicide use. This then might decrease soybean production costs, improve the CO_2_ footprint of soybean products, and minimize the potential risk of ecological and sanitary actions resulting from extensive use of fungicides (Maltby et al., 2009; Verweij et al., 2009; Wightwick et al., 2010).

### Resources of SBR Resistance in Soybean

#### *R* Genes, *R* Gene Pyramids, and Engineered *R* Genes

Analysis of soybean genotypes disclosed six dominant *R* genes conferring immunity (no visible symptoms) or resistance (reddish brown lesions and reduced sporulation) to specific *P. pachyrhizi* isolates. Those loci were referred to as *Rpp* 1–6 genes (Bromfield and Hartwig, 1980; McLean and Byth, 1980; Bromfield and Melching, 1982; Hartwig and Bromfield, 1983; Hartwig, 1986; Garcia et al., 2008; Li et al., 2012). However, *Rpp* genes provide resistance exclusively to individual *P. pachyrhizi* isolates (race-specific disease resistance). Therefore, no currently available soybean genotype would ward off all *P. pachyrhizi* isolates (Monteros et al., 2007). In addition, *Rpp* gene-mediated resistance was swiftly overcome in the field (Yorinori et al., 2005; Garcia et al., 2008). Employing recessive *R* genes might represent another approach for providing stable SBR resistance (Calvo et al., 2008). In fact, three recessive *R* genes to *P. pachyrhizi* have been identified in the soybean genotypes PI 200456, PI 224270, and BR01-18437 (Calvo et al., 2008; Pierozzi et al., 2008). These genes are now awaiting exploitation in breeding and genetic engineering for SBR resistance.

Developing elite lines and varieties requires breeders to combine traits from multiple parents, a process called gene pyramiding or stacking (Francis et al., 2012). Pyramiding *R* genes into a single genetic background is another proposed strategy for conferring soybean resistance to multiple *P. pachyrhizi* isolates (Hartman et al., 2005; Garcia et al., 2008; Lemos et al., 2011; Maphosa et al., 2012; Yamanaka et al., 2013, 2015; Bhor et al., 2014). The SBR resistant Japanese soybean cultivar Hyuuga represents a natural example of *R* gene pyramiding (Kendrick et al., 2011). In line with this finding, soybean genotypes harboring two pyramided *Rpp* genes exhibited higher SBR resistance than their ancestors containing only single *R* genes (Maphosa et al., 2012; Bhor et al., 2015). Synergistic effects were also observed when three *R* genes were bred into a single soybean genotype (Lemos et al., 2011; Yamanaka et al., 2013, 2015). Remarkably, a combination of multiple *R* genes conferred resistance to different *P. pachyrhizi* isolates from various origin (including two highly virulent strains from Brazil; Yamanaka et al., 2015). Although molecular markers facilitate breeding approaches, traditional breeding is still time consuming, and introducing unwanted traits (Salomon and Sessa, 2012). Furthermore, SBR resistance based on static *R* gene pyramids will likely be overcome upon longer use in the field (McDonald, 2014) as has been reported for other crops like wheat or barley (McDonald and Linde, 2002). Therefore, transforming expression cassettes with alternative *R* gene combinations into elite soybean lines and dynamic turnover of such lines in the field might represent a promising strategy for providing sustainable and effective SBR resistance (McDonald and Linde, 2002). However, for cloning and utilization of such multi *R* gene expression cassettes the identity of *Rpp* genes needs to be revealed. Although SBR resistance loci have been mapped to different linkage groups on various chromosomes (reviewed by Bhor et al., 2014), the identity of *Rpp* genes has remained largely unknown. One exception is represented by the NB-LRR encoding gene *Rpp4C4* that is likely responsible for *Rpp4*-mediated SBR resistance (Meyer et al., 2009).

Another possibility to enhance the resistance of soybean to SBR is to identify and exploit *R* genes conferring resistance to multiple pathogens. Several examples of such broadly active *R* genes exist in nature (Nombela et al., 2003; Narusaka et al., 2009; Atamian et al., 2012; Lozano-Torres et al., 2012). A complementary approach for broadened pathogen effector recognition uses random mutagenesis or rational design of synthetic NB-LRR immune receptors. Editing the potato NB-LRR receptor R3a at a single amino acid significantly expanded its response to *Phytophthora infestans*-derived effectors (Segretin et al., 2014). Effectively mutating the R3a orthologue I2 in tomato enhanced the response to the *P. infestans* AVR3a effector, conferred partial immunity to potato blight, and expanded the response spectrum to *Fusarium oxysporum* f. sp. *lycopersici* effectors compared to tomato plants expressing the wild-type I2 gene (Giannakopoulou et al., 2015). *R* gene engineering might also succeed in exploiting multiple *Rpp* genes for conferring an expanded response to multiple *P. pachyrhizi* isolates. *Rpp4C4* (Meyer et al., 2009) may serve for engineering such *R* gene variants by untargeted protein evolution. Furthermore, genome editing may be used for the targeted evolution of NB-LRRs. In fact, genome-wide sequence analysis predicted nearly all soybean NB-LRR-encoding genes an be targeted specifically by CRISPR/Cas9 (Xie et al., 2014).

#### Signaling Components of *R* Gene-Mediated SBR Resistance

Several studies reported differential defense responses to SBR attack in susceptible and resistant soybean genotypes. The studies included analysis of transcriptional dynamics, proteome changes, or metabolic alterations to identify loci, genes, proteins, and metabolites associated with ETI to *P. pachyrhizi* in soybean.

##### Signaling network hubs and phytohormones

Transcriptome analysis disclosed different components of *Rpp2*-mediated resistance to SBR in soybean (van de Mortel et al., 2007; Pandey et al., 2011). Of 140 candidates tested by VIGS, eleven genes clearly contributed to *Rpp2*-mediated SBR resistance. The genes encompassed *GmEDS1, GmPAD4*, and *GmNPR1*.

NPR1 is a master regulator of SAR in *Arabidopsis thaliana* and some other plants (reviewed by Fu and Dong, 2013). When overexpressed in *Arabidopsis*, rice, tobacco, or apple, NPR1 enhances resistance to infectious oomycetes, bacteria, and fungi (including obligate biotrophic fungi such as powdery mildew; Cao et al., 1998; Chern et al., 2005; Chen et al., 2012). Because of possible side effects of *NPR1* overexpression (Chern et al., 2005), such as yield reduction, the potential of this gene for generating SBR-resistant soybean varieties awaits assessment.

EDS1 and PAD4 are key regulators of several types of plant disease resistance (basal, *R* gene-mediated, and NHR). The two proteins are required for accumulation of SA, and they control various SA-dependent defense pathways (Falk et al., 1999; Jirage et al., 1999; Nawrath et al., 2002; Lipka et al., 2005; Wiermer et al., 2005; Langenbach et al., 2013; Wang et al., 2014). Because silencing of *GmEDS1* or *GmPAD4* lead to susceptibility of otherwise resistant soybean lines carrying *Rpp2*, EDS1 and PAD4 seem to control also *Rpp2*-mediated SBR resistance in soybean (Pandey et al., 2011). SA accumulation is thus likely to limit the growth and reproduction of *P. pachyrhizi* in soybean. Because *PAD4* is also required for *Arabidopsis* postinvasion NHR to *P. pachyrhizi* (Langenbach et al., 2013), SA-associated defense responses seem to be highly effective in antagonizing SBR disease. However, overexpression of SA biosynthesis genes is likely not to provide a realistic agronomical solution for SBR control because constitutive SA accumulation often causes dwarfism (Bowling et al., 1994; Li et al., 2001).

In *Arabidopsis* and soybean, *P. pachyrhizi* activates expression of JA-responsive genes at early stages of infection (Loehrer et al., 2008; Alves et al., 2015) and before actual penetration [likely by secreted *P. pachyrhizi* effectors (Campe et al., 2014)]. Since JA is considered eliciting immune responses against necrotrophic pathogens (Pieterse et al., 2012) *P. pachyrhizi* pretends being a necrotroph at initial stages of colonization. By doing so, it may circumvent effective SA-dependent defense signaling which is known to be crucial to ward off biotrophic pathogens. Thus, engineering soybean plants for the fast and robust accumulation of SA, or exploiting SA-activated downstream signaling components for resistance might be a suited strategy for providing soybean varieties resisting SBR at low risks for energetic tradeoffs.

##### Transcription factors

The importance of TFs in conferring SBR resistance became obvious when van de Mortel et al. (2007) and Schneider et al. (2011) found that TF genes are being overrepresented among genes whose expression is activated in the biphasic transcriptional response in SBR-resistant soybean genotypes harboring *Rpp2* or *Rpp3*. Amongst others, genes encoding WRKY, bHLH, and MYB TFs were activated in incompatible, but not compatible, soybean-*P. pachyrhizi* interactions. When *GmWRKY36, GmWRKY40, GmWRKY45*, and *GmMYB84* were individually silenced using VIGS, *Rpp2*-mediated SBR resistance was gone (Pandey et al., 2011). Several other studies also revealed differential expression of TFs in incompatible or compatible soybean-*P. pachyrhizi* interactions (Panthee et al., 2009; Morales et al., 2013; Aoyagi et al., 2014). In fact, there seems to be considerable overlap of TF activity in *Rpp2, Rpp3*, and *Rpp4*-mediated soybean disease resistance (Morales et al., 2013). Therefore, these TFs seem to be excellent candidates for engineering SBR resistance. However, manipulation of TF balance may affect agronomic traits because TFs regulate a diverse array of loci.

In another approach, Cooper et al. (2011) compared nuclear proteome changes in a resistant vs. susceptible genotype at 24 h after inoculation with *P. pachyrhizi*. Their analysis disclosed more than 200 proteins that specifically accumulated in the nucleus of SBR-resistant soybean plants harboring *Rpp1* (Cooper et al., 2011). Silencing two predicted soybean TFs (Glyma14g11400, PHD superfamily and Glyma12g30600, zinc finger TF) via VIGS partially compromised *Rpp1*-conferred SBR resistance (Cooper et al., 2013). Similarly, Bencke-Malato et al. (2014) demonstrated that accumulation of mRNA transcripts for several WRKY TFs was faster and more robust in a resistant than susceptible soybean accession. Consistently, the simultaneous silencing of four identified *WRKY* genes rendered soybean plants more susceptible to SBR disease. Because the authors did not succeed in producing *WRKY*-overexpressing soybean lines (Bencke-Malato et al., 2014), the potential of *WRKY* overexpression for providing SBR resistance to susceptible soybean genotypes remained unclear.

##### Secondary metabolism

Plants can halt or slow down infection by constitutive or inducible accumulation of antimicrobial and/or cell wall-fortifying secondary metabolites (Chiang and Norris, 1983; Hahlbrock and Scheel, 1989; Chang et al., 1995; Dixon et al., 2002; Boerjan et al., 2003; La Camera et al., 2004; Vogt, 2010). Secondary metabolites also contribute to the outcome of the soybean–*P. pachyrhizi* interaction. Daidzein, genistein, and glyceollin are isoflavonoids that accumulate in both resistant and susceptible soybean genotypes upon *P. pachyrhizi* infection (Lygin et al., 2009). Glyceollin efficiently reduces *P. pachyrhizi* uredospore germination *in vitro* (Lygin et al., 2009). Further evidence for a role of phytoalexins in SBR resistance was provided by Bilgin et al. (2009). The authors disclosed that SBR resistance in a *Glycine tomentella* accession correlated with the presence of a flavonoid that also inhibited *P. pachyrhizi* spore germination (Chung and Singh, 2008). The high potential of phytoalexins in defeating SBR is further supported by medicarpin accumulating in *P. pachyrhizi*-infected *Medicago truncatula*, a non-host of *P. pachyrhizi*. Consistently, medicarpin inhibits *P. pachyrhizi* spore germination (Ishiga et al., 2015). Providing such comparative large-scale metabolic profiles from resistant vs. susceptible soybean varieties, or other SBR-resistant species would likely identify more secondary metabolites inhibiting SBR. Genes in their biosynthesis pathways could be used to engineer SBR resistance in transgenic soybean. Alternatively, the compound(s) themselves could serve as natural fungicides in spray application, especially if they can be produced at low costs and in sufficient quantities for use in agriculture. In a variety of studies, genes in the phenylpropanoid and flavonoid metabolism were overrepresented when analyzing the transcriptional response of infected soybean genotypes with SBR resistance (van de Mortel et al., 2007; Choi et al., 2008; Panthee et al., 2009; Schneider et al., 2011). Overall, activation of these genes was faster and stronger in SBR-resistant accessions than in susceptible ones (van de Mortel et al., 2007; Schneider et al., 2011). Functional evidence for the importance of phenylpropanoid pathway genes in soybean’s SBR resistance was provided by Pandey et al. (2011). The authors demonstrated that silencing of soybean phenylalanine ammonia-lyase (*GmPAL*) or *O*-methyl transferase1 (*GmOMT1*) compromised *Rpp2*-mediated SBR resistance. *OMT1* silencing also partially impaired *Rpp1*-mediated SBR resistance (Cooper et al., 2013) and significantly decreased lignin content (Pandey et al., 2011). The latter result points to an important role of lignification in rejecting SBR.

#### Susceptibility Genes and Effector Targets

Different from dominant *R* genes conferring effective, but exclusively race-specific and non-durable resistance (Yorinori et al., 2005; Garcia et al., 2008), the loss of functional *S* genes can eventually provide durable disease resistance (Pavan et al., 2010; Gawehns et al., 2013). For example, in barley absence of the *S* gene *Mlo* results in an incompatible interaction with *Blumeria graminis* f. sp. *hordei* that resembles NHR (Humphry et al., 2006). *S* genes function either as susceptibility factors or suppressors of plant defense. Thus they are potential targets of fungal effectors. Consistent with this assumption knocking out *S* genes leads to recessive resistance with effectivity to multiple races of a given pathogen (Pavan et al., 2010). This type of resistance is very stable. The resistance of plants harboring recessive alleles of *Mlo* (barley) or *eIF4E* (pepper) has not been overcome in the field for 30–50 years (Lyngkjær et al., 2000; Kang et al., 2005). Breeding for *S* gene variants insensitive to manipulation by pathogen effectors therefore has huge potential for durable, broad-spectrum disease resistance; although loss-of-function mutations in *S* genes may be associated with pleiotropic detrimental effects (Büschges et al., 1997).

Soybean *S* genes to SBR have not been identified so far. However, several approaches might identify potential *S* gene alleles for SBR resistance in soybean. Because most *S* genes of agricultural value were identified in screens for recessive resistance in wild species of plant (Bai et al., 2005), searching for such a resistance in wild *Glycine* might similarly provide genetic resources for breeding or engineering SBR resistance in *G. max*.

Another option for identifying soybean *S* genes to SBR is via sequence homology search to known *S* genes. Functional analysis can be done using, for example, soybean insertion mutants (Mathieu et al., 2009), performing VIGS (Zhang and Ghabrial, 2006; Zhang et al., 2010, 2013; Pandey et al., 2011), TILLING (Cooper et al., 2008), or applying targeted genome editing techniques such as CRISPR/Cas9 (Jacobs et al., 2015). However, currently only one gene [the Cys(2)His(2) zinc finger TF palmate-like pentafoliata1, *PALM1*] that would classify as an SBR *S* gene is known from *M. truncatula* (Uppalapati et al., 2012). Alternatively, fungal effectors might serve as guides to identify novel *S* genes in soybean and other plants since several effectors of bacteria, fungi, or oomycetes were shown to target plant *S* genes (reviewed by Gawehns et al., 2013). Although various analyses identified stage-specific rust proteins that might have bona-fide effector function (Loehrer and Schaffrath, 2011; Stone et al., 2012; Link et al., 2014), their role as virulence factors awaits functional confirmation. Identification of effector proteins and corresponding *S* gene targets was likely hampered by missing *P. pachyrhizi* genome information (Loehrer et al., 2014). Transformation protocols enabling generation of *P. pachyrhizi* knockout mutants are also missing.

Another approach for identifying *S* gene alleles conferring SBR resistance is via screening of mutagenized soybean populations for loss-of-susceptibility mutants. The tetraploid nature of the soybean crop and the potential existence of multiple *S* gene copies might hamper this approach. Because 12 duplicated copies of a given DNA region might be present in the soybean genome (Cannon and Shoemaker, 2012), mutagenesis-induced phenotypic variation might be buffered by gene redundancy (Bolon et al., 2014). However, fast neuron irradiation recently provided more than 27,000 unique soybean mutants with significant phenotypic variation (Bolon et al., 2011, 2014). The mutants may facilitate genetic screens for loss of SBR susceptibility mutants with interesting resistance phenotypes similar to the *M. truncatula irg1* mutant (Ishiga et al., 2015). Identified *S* gene alleles for SBR resistance in soybean might be engineered in elite soybean lines via genome editing (Jacobs et al., 2015).

#### Genes Providing Quantitative SBR Resistance or Tolerance

Forward genetic screens using activation-tagged soybean plants (Mathieu et al., 2009) could identify genes and loci that quantitatively contribute to SBR resistance. Genes and loci for SBR resistance can potentially also be found exploiting fungal effectors targeting proteins with a role in apoplastic immunity [e.g., the *Ustilago maydis* effector Pit2 targets maize apoplastic cysteine proteases (Mueller et al., 2013)].

PDR to SBR is found in ‘slow rusting’ soybean accessions such as SRE-Z-11A, SRE-Z-11B, and SRE-Z-15A (Tukamuhabwa and Maphosa, 2010). These genotypes can potentially provide useful genes and loci for quantitative SBR resistance. PDR is characterized by low infection frequency, long-lasting latency, small lesions, and reduced spore production per uredinium. Thus, PDR reduces SBR epidemics (Tukamuhabwa and Maphosa, 2010). Since PDR is polygenic and effective to multiple pathogen races (Long et al., 2006), identification, and transfer of genes from partially resistant to susceptible soybean varieties might provide only partial but durable resistance to diverse *P. pachyrhizi* isolates. Because of PDR’s polygenic nature and the time-consuming process for selecting partially resistant progeny, such soybean varieties have not attracted much attention as sources for SBR resistance in the past (Hartman et al., 2005).

Besides soybean genotypes with partial resistance, SBR-tolerant accessions also have not been a subject of molecular research. Although susceptible to SBR, these genotypes do better tolerate the presence of *P. pachyrhizi* and produce reasonably high yield even when severely infected. Yield may increase by 30–60% using SBR-tolerant varieties in the presence of *P. pachyrhizi* (Tukamuhabwa and Maphosa, 2010). Furthermore, planting tolerant varieties does not pose selection pressure on *P. pachyrhizi*, thus minimizing the risk of selecting adapted pathogen races (Arias et al., 2008). However, SBR disease tolerance of a given soybean accession is assessed with respect to its yield capacity. This requires multi-site field trials and hinders evaluation of a genotype’s tolerance and commercial value at small scale laboratory conditions (Tukamuhabwa and Maphosa, 2010). Nonetheless, identification of genes for SBR tolerance using, e.g., comparative transcriptome or proteome analysis, may enable provision of soybean varieties with capacity for enhanced yield at high SBR pressure.

#### Antimicrobial Peptides

AMPs can provide disease resistance to plants (Rahnamaeian, 2011). However, AMPs did not serve to fight SBR so far. Brand et al. (2012) introduced a method for the identification and employment of putative AMPs encrypted in soybean protein sequences. This approach was meant to provide an alternative to transgenic approaches that expressed AMPs from other organisms. Using *in situ* assays, Brand et al. (2012) found that IAPs conferred SBR resistance in a manner similar to AMPs from *Phyllomedusa* ssp. (dermaseptin SI) or *Drosophila melanogaster* (penetratin) when co-incubated with fungal uredospores on susceptible soybean leaves. In addition, soybean plants expressing a putative antimicrobial fragment of the *G. max* D-myo-inositol 3-phosphate synthase [IAP gb|ABM17058.1| (213–231)] showed enhanced resistance to *P. pachyrhizi* (Brand et al., 2012). These findings illustrate the feasibility of trans- or cisgenic AMP expression for SBR resistance.

### Alternative Sources of SBR Resistance

#### Wild *Glycine* Species and Other Alternative Hosts

Wild perennial *Glycine* species might serve as valuable resources of germplasm for SBR resistance. This is because *Glycine clandestina, Glycine canescens, Glycine tabacina, Glycine tomentella*, and *Glycine argyrea* all display pathotype-specific resistance to *P. pachyrhizi* (Burdon and Speer, 1984; Burdon, 1987, 1988; Jarosz and Burdon, 1990). In *G. clandestina, G. canescens*, and *G. argyrea* differential SBR resistance phenotypes are linked to presence or absence of single or multiple (pyramided) *R* genes (Burdon and Speer, 1984; Burdon, 1987, 1988; Jarosz and Burdon, 1990). The resistance of *G. tomentella* accession PI 441001 to *P. pachyrhizi*, however, was associated with accumulation of an antifungal flavonoid inhibiting *P. pachyrhizi* spore germination (Chung and Singh, 2008). Because Singh and Nelson (2015) obtained fertile SBR-resistant plants from crosses of *G. max* and *G. tomentella*, transfer of *R* genes from wild perennial species to commercial soybean varieties via intersubgenic hybridization seems to be a powerful strategy for SBR resistance. The novel hybrid plant is still to be analyzed for its yield and resistance to multiple *P. pachyrhizi* isolates which will disclose the commercial value of the hybrid.

Other SBR resistance traits are present in *G. soja*. The species is closer related to *G. max* than its above mentioned wild perennial relatives (Bromfield, 1984). However, because of presence of undesired traits, generating hybrids for commercialization using *G. soja* or the wild, perennial *Glycine* species will likely require elaborate backcrossing and selection. Identifying the genetic basis of SBR resistance in wild species followed by engineered transfer of genes and/or traits to elite varieties might represent an alternative, more promising strategy for SBR resistance. The approach circumvents the drawbacks associated with hybridization strategies. However, only few attempts (e.g., Soria-Guerra et al., 2010) identified gene candidates to condition SBR resistance in wild *Glycine* species.

Kudzu (*Pueraria lobata*) is a leguminous weed that hosts *P. pachyrhizi* and could provide traits for SBR resistance. Genetic variation is high among different kudzu populations but low within a same population (Sun et al., 2005). As a consequence, individual kudzu plants are resistant/immune or susceptible to diverse *P. pachyrhizi* isolates (Bonde et al., 2009). In a kudzu genotype with immunity to SBR the early abrogation of *P. pachyrhizi* infection correlated with cell wall appositions and cell death in the leaf epidermis (Jordan et al., 2010). This finding suggests presence of early, effective defense responses in immune kudzu genotypes. Big differences in the response to *P. pachyrhizi* infection were also seen in several other legume species (Slaminko et al., 2008). *Vigna adenantha* PI 312898, for instance, is immune to SBR as are individual bean (*Phaseolus vulgaris*) cultivars (Miles et al., 2007; Souza et al., 2014). However, lack of genomic information and low genetic accessibility of alternative *P. pachyrhizi* hosts impede candidate gene identification and gene transfer.

#### Non-host Plants

Over the past decade, employing non-host plants has become a promising approach for identifying resistance traits. Due to the pervasive nature of NHR, the strategy explores a vast genetic resource. NHR is a multi-layered, complex type of plant disease resistance that shares signaling and defense mechanisms with host resistance (Schulze-Lefert and Panstruga, 2011). Classification of a given plant species as a host or non-host can be difficult because there seems to be a gradual continuum from host to non-host with many intermediate resistances (Bettgenhaeuser et al., 2014). Exploring the molecular basis of this variety of resistances and pyramiding underlying genes and loci in the soybean crop may represent a powerful approach for SBR resistance and provide an alternative to chemical fungicides and traditional breeding.

*Arabidopsis* and *M. truncatula* are the best described plants in terms of NHR to *P. pachyrhizi*. Since *P. pachyrhizi* does not produce macroscopic symptoms on any of 28 wild-type accessions tested, *Arabidopsis* can be considered a true non-host for *P. pachyrhizi* (Loehrer et al., 2008). Although initial stages of *P. pachyrhizi* development are identical on *Arabidopsis* and soybean, proliferation of *P. pachyrhizi* hyphae into the leaf mesophyll is rare in *Arabidopsis* (Loehrer et al., 2008). To determine the molecular basis of the preinvasion resistance to *P. pachyrhizi* in this plant, Loehrer et al. (2008) used *Arabidopsis* mutants with known compromised resistance to other non-adapted fungal pathogens. Colonization of the mesophyll occurred in *Arabidopsis penetration* mutant *pen1, pen2*, and *pen3*. However, despite hyphal growth and rarely observed haustoria in the mesophyll of *pen* mutants, the fungus failed to successfully colonize the plant. It also did not complete its life cycle, indicative of functional postinvasion resistance to *P. pachyrhizi* in these mutants. The postinvasion resistance was compromised in the *Arabidopsis* triple mutant *pen2 pad4 sag101* in which *P. pachyrhizi* frequently developed haustoria (Langenbach et al., 2013). However, extensive mesophyll colonization and sporulation did not occur in any *Arabidopsis* mutants tested.

To identify components of *Arabidopsis* postinvasion resistance to *P. pachyrhizi*, Langenbach et al. (2013, 2016) performed comparative transcriptional profiling of genes specifically activated upon *P. pachyrhizi* infection in *pen2* (a mutant with intact postinvasion resistance) but not *pen2 pad4 sag101* (with compromised postinvasion resistance). The screen identified BRIGHT TRICHOMES 1 (BRT1), an UDP-glycosyltransferase in the phenylpropanoid metabolism. Postinvasion resistance to *P. pachyrhizi* was impaired in the *pen2 brt1* double mutant. In this genotype the fungus developed more haustoria than in *pen2*. Since *brt1* mutants were not affected in preinvasion SBR resistance (Langenbach et al., 2013), BRT1 seems to specifically contribute to postinvasion NHR to the disease.

To identify more genes that function in *Arabidopsis* NHR to SBR, Langenbach et al. (2016) searched for genes co-regulated with *BRT1*. Upon confirming the genes’ importance in *Arabidopsis* postinvasion resistance, the authors expressed these genes in soybean. Four so-called postinvasion-induced NHR genes (*PINGs*) indeed reduced SBR disease severity. The supposed function of individual PING proteins is quite diverse and includes an EARLI4-like phospholipase (PING4), a group I receptor-like kinase (PING5), a GDSL-like lipase (PING7), and a germin-like protein (PING9). The exact mode of action of PINGs in conferring resistance to *P. pachyrhizi* has remained elusive (Langenbach et al., 2016). However, the study discloses that interspecies gene transfer is a promising strategy for conferring SBR resistance to soybean. Gene donor and receiver plant obviously do not need to be closely related, although it is likely that the successful transfer of a protein’s function from one species to another implies conservation or convergence of its physiological environment (e.g., signaling networks). Thus, employing phylogenetically related non-hosts might further enhance the success of interspecies NHR gene transfer as a means for SBR resistance. Because *P. pachyrhizi* infects many plants, non-hosts to the fungus are rare, especially in the legume family of plants. *M. truncatula* is the only reported leguminous non-host as sporulation of *P. pachyrhizi* has not been observed on this plant (Uppalapati et al., 2012; Ishiga et al., 2015). The former authors did a forward genetic screen to identify *M. truncatula* mutants with altered resistance to *P. pachyrhizi*. Because of its diploid genome, *M. truncatula* is better suited for forward genetic screening than the allopolyploid soybean crop (Gill et al., 2009). Furthermore, there is highly conserved microsynteny between soybean and *M. truncatula* (Yan et al., 2003). The screen by Uppalapati et al. (2012) identified an *inhibitor of rust germ tube differentiation* (*irg*)*1* mutant on which *P. pachyrhizi* failed to promote preinfection structures. It turned out that the loss of abaxial epicuticular wax crystals and the reduced surface hydrophobicity inhibited fungal development on *irg1* (Uppalapati et al., 2012). The mutation was mapped to *PALM1* encoding a Cys(2)His(2) zinc finger TF controlling the expression of genes involved in long-chain fatty acid biosynthesis and transport (Uppalapati et al., 2012).

To further investigate the role of surface hydrophobicity or epicuticular waxes on *P. pachyrhizi* development, Ishiga et al. (2013) recorded the fungal transcriptome during germination on a hydrophobic surface (glass slides coated with epicuticular wax from wild-type plants and *irg1*/*palm1* mutants) and on the leaf surface of *M. truncatula* wild-type plants and the *irg1/palm1* mutant. They found expression of kinase family genes was activated on the hydrophobic surface and on the *M. truncatula* wild type but not on *irg1/palm1*. This result suggested that leaf hydrophobicity or epicuticular waxes may trigger expression of *P. pachyrhizi* genes involved in pre-penetration structure formation (Ishiga et al., 2013). Importance of cutin or cuticular waxes to both, germination and appressoria formation has also been reported for other fungal pathogens of plants (Mendoza-Mendoza et al., 2009; Hansjakob et al., 2011; Weidenbach et al., 2014). Further characterization of the *irg1/palm1* mutant may help better understand asymmetric epicuticular wax loading on leaf surfaces and its importance to plant-pathogen interactions. Additionally, identifying *IRG1/PALM1* orthologues and/or modifying epicuticular wax composition in soybean might be useful to conferring resistance to *P. pachyrhizi*.

Transcriptome analysis of the *M. truncatula–P. pachyrhizi* interaction revealed induction of many genes in the phenylpropanoid, flavonoid, and isoflavonoid pathways (Ishiga et al., 2015). Accompanying metabolome studies disclosed accumulation of the isoflavonoid derivative medicarpin and its intermediates in *P. pachyrhizi*-inoculated plants. Because medicarpin inhibited the germination and differentiation of *P. pachyrhizi* uredospores *in vitro* (Ishiga et al., 2015), the phytoalexin might contribute to NHR to *P. pachyrhizi* in *M. truncatula*. Various studies with *P. pachyrhizi* hosts also pointed to a role of phytoalexins in the interaction of plants with the fungus (Chung and Singh, 2008; Lygin et al., 2009). As the expression of genes in the secondary metabolism is strongly affected upon *P. pachyrhizi* infection in soybean (van de Mortel et al., 2007; Choi et al., 2008; Panthee et al., 2009; Schneider et al., 2011), secondary metabolites seem to be crucial to both host resistance and NHR to SBR.

### RNA Interference and Host-Induced Gene Silencing

Another option for controlling SBR is by using RNAi to specifically silence essential *P. pachyrhizi* genes. HIGS, a specific RNAi technique, provided protection from sucking insects, nematodes, fungi, oomycetes, bacteria, and viruses (Koch and Kogel, 2014). To our knowledge there is not a single report on the application of HIGS in soybean for fighting SBR or other fungal diseases. However, knockdown of nematode genes by siRNAs expressed in soybean was demonstrated (Steeves et al., 2006; Li et al., 2010; Niu et al., 2012; Youssef et al., 2013). Moreover, the successful silencing of fungal genes, including those of the rust fungi *Puccinia striiformis, P. triticina*, and *P. graminis* in other crops (Yin et al., 2010; Panwar et al., 2013) is testament to the huge potential of this approach for fighting SBR. Various stage-specifically expressed fungal genes that may represent potential HIGS targets (e.g., genes encoding putative effectors like HESPs, kinase family proteins, cell wall degrading enzymes, metabolism-linked genes, succinate dehydrogenase, etc.) have already been identified in *P. pachyrhizi* (Posada-Buitrago and Frederick, 2005; Stone et al., 2012; Tremblay et al., 2012, 2013; Ishiga et al., 2013; Link et al., 2014). Since external application of dsRNAs has proven effective for the control of insect pests (Hunter et al., 2012), this approach might present a non-transgenic alternative to HIGS-mediated SBR control.

### Biocontrol

*In vitro* studies and greenhouse and field trials reported protection by beneficial microbes with antagonistic properties to *P. pachyrhizi*. The fungus *Simplicillium lanosoniveum* preferentially colonizes *P. pachyrhizi* uredinia on infected soybean leaves and thereby significantly reduces SBR development in the field (Ward et al., 2012). Similarly, Kumar and Jha (2002) observed hypertrophy and shrinkage of *P. pachyrhizi* uredospores when colonized with *Trichothecium rosae*. Moreover, several strains of *Bacillus* spp. reduce SBR severity (Dorighello et al., 2015). One *Bacillus* strain that is the active ingredient in the organically approved commercial fungicide Ballad^®^ provides SBR control. Besides antagonistic organisms, plant volatiles, such as farnesyl-acetate, can be used for biocontrol of SBR (Mendgen et al., 2006). Same is true for coffee oil and essential oils from *Hyptis marrubioides, Aloysia gratissima*, and *Cordia verbenacea* which suppressed spore germination *in vitro* and reduced SBR severity under greenhouse and/or field conditions (da Silva et al., 2014; Dorighello et al., 2015). Moreover, acibenzolar-*S*-methyl treatment or soil application of silicon reduced SBR severity on soybean leaves (da Cruz et al., 2013). Silicon most likely acts in two ways. First, it establishes a physical penetration barrier when deposited in the subcuticular layer and second, it primes plants for enhanced defense (Ma and Yamaji, 2006; da Cruz et al., 2013). Furthermore, soil application of saccharin and shale water were reported to induce SBR resistance in soybean (Srivastava et al., 2011; Mehta et al., 2015). These examples illustrate the potential of SBR biocontrol. However, the cost-benefit ratio and feasibility of field scale biocontrol needs to be determined to estimate the actual agronomic value of such approaches.

## Conclusion

*Phakopsora pachyrhizi* is the causal agent of SBR and thus a major threat to global soybean production. Novel compounds in the SDHI class of fungicides hold promise for successful SBR control in the upcoming years, but *P. pachyrhizi* is likely to become increasingly insensitive to SDHI action as it has been observed for DMI and QoI fungicides. Similarly, the SBR resistance conferred by individual *R* genes was swiftly overcome in the field, but the pyramiding (stacking) of known and yet to be identified *R* genes might overcome traditional *R* gene inefficacy. Exploiting pathway components for the major plant defense hormones, SA and JA, seems not to be a realistic option for SBR control because component overexpression often impairs plant growth and yield. By contrast, transcription coactivator utilization could have huge potential but their efficacy for effective SBR control is still awaiting assessment in both the lab and field. Synthetic biology approaches to engineer *R* genes and phytoalexin biosynthesis pathways are promising, especially because several phytoalexins antagonize *P. pachyrhizi* both *in vitro* and *in situ*. Loss or elimination of *S* genes also is promising for SBR control but this approach has rarely been followed up. Same is true for the exploitation of soybean accessions with tolerance or PDR to SBR. Though promising, their potential for SBR control is currently unclear. Wild *Glycine* species, alternative *P. pachyrhizi* hosts, and especially non-host plants are promising sources of germplasm for SBR resistance while AMPs, RNAi/HIGS, and biocontrol approaches hold promise for sustainable soybean production in the future. It seems that an integrated approach exploiting different measures is likely to provide the best possible means for the effective control of SBR.

## Author Contributions

CL, RC, SB, and AM contributed various chapters to the article. CL composed the review. CL, AM, and UC thoroughly reviewed the consecutive manuscript drafts.

## Conflict of Interest Statement

Author RC is an employee of BASF Plant Science Company GmbH.

## Abbreviations

AMP: Antimicrobial peptide
CRISPR: Clustered regularly interspersed short palindromic repeats
DMI: Demethylation inhibitor
dsRNA: Double stranded RNA
ETI: Effector-triggered immunity
FRAC: Fungicide Resistance Action Committee
HESP: Haustoria-expressed secreted protein
HIGS: Host-induced gene silencing
IAP: Intragenic antimicrobial peptide
JA: Jasmonic acid
MDR: Muldidrug resistance
NB-LRR: Nucleotide-binding leucine-rich repeat
NHR: non-host resistance
PDR: Partial disease resistance
QoI: Quinone outside inhibitor
*R* gene: Resistance gene
RNAi: RNA interference
*Rpp*: Resistance to *P. pachyrhizi*
*S* gene: Susceptibility gene
SA: Salicylic acid
SAR: Systemic acquired resistance
SBR: Asian soybean rust
SDHI: Succinate dehydrogenase inhibitor
siRNA: Small interfering RNA
TF: Transcription factor
TILLING: Targeting induced local lesions in genomes
VIGS: Virus-induced gene silencing
